# Stem cell treatment of degenerative eye disease^☆^

**DOI:** 10.1016/j.scr.2015.02.003

**Published:** 2015-05

**Authors:** Ben Mead, Martin Berry, Ann Logan, Robert A.H. Scott, Wendy Leadbeater, Ben A. Scheven

**Affiliations:** a Neurotrauma Research Group, Neurobiology Section, School of Clinical and Experimental Medicine, University of Birmingham, B15 2TT, UK; b School of Dentistry, University of Birmingham, B4 6NN, UK

**Keywords:** ADSCs, adipose-derived stem cells, AMD, age-related macular degeneration, BDNF, brain-derived neurotrophic factor, BMSCs, bone marrow-derived stem cells, CNS, central nervous system, CNTF, ciliary neurotrophic factor, DPSC, dental pulp stem cells, EGF, epidermal growth factor, ERG, electroretinogram, ESCs, embryonic stem cells, FGF, fibroblast growth factor, GDNF, glial cell line-derived neurotrophic factor, GFAP, glial fibrillary acidic protein, iPSCs, induced pluripotent stem cells, *ivit*, intravitreal, MSC, mesenchymal stem cells, mTOR, mammalian target of rapamycin, NGF, nerve growth factor, NSCs, neural stem cells, NT-3, neurotrophin-3, NTFs, neurotrophic factors, ONL, outer nuclear layer, RCS, Royal College of Surgeons rats, RGC, retinal ganglion cell, RPE, retinal pigment epithelial cells, SCI, spinal cord injury, TBI, traumatic brain injury, TrK, tropomyosin related kinase, VEGF, vascular endothelial growth factor.

## Abstract

Stem cell therapies are being explored extensively as treatments for degenerative eye disease, either for replacing lost neurons, restoring neural circuits or, based on more recent evidence, as paracrine-mediated therapies in which stem cell-derived trophic factors protect compromised endogenous retinal neurons from death and induce the growth of new connections. Retinal progenitor phenotypes induced from embryonic stem cells/induced pluripotent stem cells (ESCs/iPSCs) and endogenous retinal stem cells may replace lost photoreceptors and retinal pigment epithelial (RPE) cells and restore vision in the diseased eye, whereas treatment of injured retinal ganglion cells (RGCs) has so far been reliant on mesenchymal stem cells (MSC). Here, we review the properties of non-retinal-derived adult stem cells, in particular neural stem cells (NSCs), MSC derived from bone marrow (BMSC), adipose tissues (ADSC) and dental pulp (DPSC), together with ESC/iPSC and discuss and compare their potential advantages as therapies designed to provide trophic support, repair and replacement of retinal neurons, RPE and glia in degenerative retinal diseases. We conclude that ESCs/iPSCs have the potential to replace lost retinal cells, whereas MSC may be a useful source of paracrine factors that protect RGC and stimulate regeneration of their axons in the optic nerve in degenerate eye disease. NSC may have potential as both a source of replacement cells and also as mediators of paracrine treatment.

## Introduction

The loss of retinal neurons, their connections and supporting glia in ocular degenerative diseases causes permanent blindness, principally because lost photoreceptors and retinal ganglion cells (RGCs) are not replaced and RGC axons fail to regenerate (Berry et al., 2008). Clinically, there are neither neuroprotective nor axogenic therapies available that restore lost visual system connectivity in retinal degenerative disease and translatable techniques for the replacement of lost RGC and photoreceptors are in their infancy. The retina is classified as central nervous system (CNS) tissue and the characteristics of its regenerative response are shared by other CNS tissues, including the brain and spinal cord.

Stem cell treatments developed as therapies for retinal degeneration fall into two broad categories: stem cells from (1), sources exogenous to the retina including mesenchymal stem cells (MSC) neural stem cells (NSCs) and embryonic/induced pluripotent stem cells (ESCs/iPSCs); and (2), endogenous retinal stem cells such as Müller glia (Ooto et al., 2004; Reichenbach and Bringmann, 2013), ciliary epithelia-derived stem cells (Ahmad et al., 2000; Tropepe et al., 2000) and retinal pigment epithelial (RPE) stem cells.

Potential non-retinal-derived adult stem cell based strategies being developed to treat retinal degeneration include NSC (McGill et al., 2012; Lu et al., 2013) and MSC derived from either bone marrow (BMSC) (Yu et al., 2006; Johnson et al., 2010; Levkovitch-Verbin et al., 2010), adipose tissues (ADSC) (Tsuruma et al., 2014) or dental pulp (DPSC) (Mead et al., 2013). MSC predominantly provide trophic support for the neuroprotection and axon regeneration of damaged retinal cells either directly through the secretion of neurotrophic factors (NTFs) (Johnson et al., 2010; Johnson et al., 2013; Mead et al., 2013) or possibly indirectly after stimulation of endogenous retinal cells (Lee et al., 2012) which, when activated, could provide additional paracrine support and/or effect cell replacement. There is no evidence that ESCs/iPSCs provide substantial paracrine support, but they do seem to be able to replace degenerating photoreceptors and RPE cells (Carr et al., 2009b; Lamba et al., 2009). NSCs directly differentiate into neural and glial phenotypes after transplantation into spinal cord injury (SCI) and traumatic brain injury (TBI) sites (Jeong et al., 2003; Lu et al., 2012). They also secrete trophic factors (Lu et al., 2003) and, although limited work has been performed in the eye with NSC, may have potential for both the neuroprotection and replacement of retinal neurons, including RGC. The differential efficiency of NSC/MSC/ESC/iPSC to perform these disparate tasks is the key to identifying the phenotype most fitted to provide the optimal safe therapy for retinal disease.

Of the endogenous retinal stem cells, Müller glia have been induced to dedifferentiate into retinal progenitors which can then transform into multiple retinal phenotypes including photoreceptors in the photoreceptor-damaged eye (Osakada et al., 2007; Liu et al., 2013). Ciliary epithelial-derived stem cells are self-renewing, multipotential retinal progenitor cells found in the pigmented ciliary epithelium of the retina (Xu et al., 2007), some of which differentiate in vitro into rhodopsin^+^ photoreceptors (Ballios et al., 2012; Clarke et al., 2012; Del Debbio et al., 2013). The RPE layer generates new retina in some animals (Fischer, 2005) and, in humans, contains a small population of stem cells that can mature into new RPE cells as well as cells with a neuronal phenotype (Salero et al., 2012). Whilst manipulation (Yu et al., 2014) and transplantation (Chacko et al., 2003; Canola et al., 2007) of endogenous retinal stem cells have the potential to treat retinal degeneration, their mechanism of action is largely restricted to RPE and photoreceptor replacement with RGC replacement proving more refractory to such strategies.

There are currently many clinical trials ongoing which aim to test the safety and efficacy of stem cell transplantation in the eye (Table 1). This review focuses on the potential of non retinal-derived stem cells, in particular NSC, BMSC, ADSC, DPSC and ESC/iPSC for the treatment of traumatic and degenerative eye disease and, where relevant, inter-relates some findings from stem cell research in the spinal cord and brain. Much overlap exists regarding the mechanisms and efficacy of ESC and iPSC and is therefore discussed together, readers are directed towards the following reviews for specific discussion on ESC (Reynolds and Lamba, 2013) and iPSC (Wright et al., 2014) for the treatment of the retina. Readers are also referred to the following articles discussing treatments using MSC not discussed in this review, such as umbilical blood-derived MSC (Zwart et al., 2009; Chen et al., 2013), as well as endogenous retinal stem cells (Yu et al., 2014).

## Ntf-mediated effects of stem cells

### Retinal cell degeneration

The extensive literature on NTF-mediated neuroprotection has been reviewed by Barde (1989), Sofroniew et al. (2001), Jones et al. (2001) and Morgan-Warren et al. (2013). After uptake by axons innervating distant neuronal targets, NTFs are retrogradely transported to somata (Dawbarn and Allen, 2003) where they are neuroprotective. During development, neurons that fail to innervate their targets are starved of these survival signals and die by apoptosis (Butowt and von Bartheld, 2003). Many adult axotomised neurons also atrophy and die after disconnection from target-derived NTF, but the viability of neurons with collaterals proximal to the transection site is protected by a supply of NTF from spared innervated targets and from local glia (Dougherty et al., 2000; Faulkner et al., 2004). Since axon collaterals are absent in the optic nerve, RGCs are exquisitely sensitive to optic nerve damage, so that approximately 40% die within 7 days (Ahmed et al., 2011) and 90% are lost by 14–21 days (Mey and Thanos, 1993; Berkelaar et al., 1994). RGC loss detailed above is of relevance to diseases such as glaucoma and traumatic optic neuropathy.

**Figure 1:**
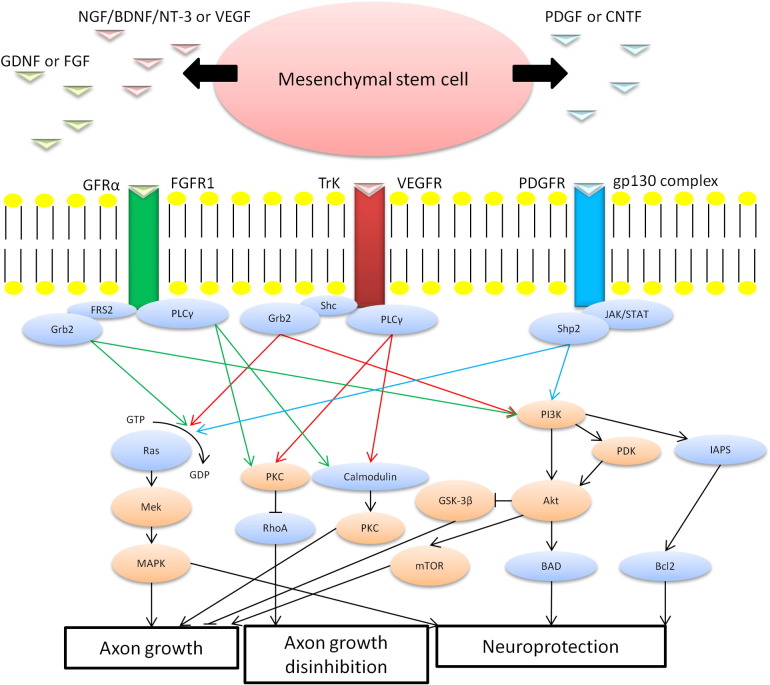
A schematic diagram showing the proposed mechanism by which MSCs exert their neurotrophic effects on the injured CNS, including the retina, through secretion of NGF, BDNF, NT-3, CNTF GDNF, VEGF, FGF and PDGF which engage TrK A, B and C, CNTFα, GFRα, VEGFR, FGFR1 and PDGFR receptors, respectively, leading to the activation of intracellular pathways for axon growth, axon growth disinhibition and neuroprotection, accounting for the functional recovery seen in animals receiving MSC transplants after CNS/retinal injury. Some ligand-receptor interactions lead to the activation of the same signalling pathways (abbreviations: BAD, bcl-2-associated death promoter; Bcl2, B-cell lymphoma 2; FGFR1, fibroblasts growth factor receptor 1; FRS2, fibroblast growth factor receptor substrate 2; GFRα, GDNF family receptor alpha; Grb2, growth factor receptor-bound protein 2; GSK-3β, glycogen synthase kinase-3β; IAPS, inhibitor of apoptosis; MAPK, mitogen-activated protein kinase; Mek, mitogen-activated protein kinase; PDK, phosphoinositide-dependant kinase; PKC, protein kinase C; PLCγ, phospholipase C-gamma).

The failure of adult CNS neurons to regenerate damaged axons is attributed to suppression of intrinsic axogenic machinery, the paucity of NTF essential for axon growth cone advance (Berry et al., 2008) and the presence of axon growth inhibitory factors (Richardson et al., 1980; Sandvig et al., 2004) mediating growth cone collapse although the relative importance of these differing factors is debatable. The neurotrophins nerve growth factor (NGF), brain-derived neurotrophic factor (BDNF) and neurotrophin-3 (NT-3) phosphorylate tyrosine residues (Dawbarn and Allen, 2003) after binding to the tropomyosin related kinase (TrK) receptor and promote RGC survival and axon growth (Berry et al., 2008) by activating intracellular signalling pathways (MAPK/PI3K/PKC; Fig. 1), whilst ciliary neurotrophic factor (CNTF) activates the JAK pathway after binding to the heterotrimeric gp130 receptor complex and signal through phosphoinositide-3-kinase (PI3K)/protein kinase B (Akt) to activate the serine–threonine kinase mammalian target of rapamycin (mTOR), to promote axogenic protein synthesis and inhibit glycogen synthase kinase-3β (GSK3β) which, amongst other roles, regulates growth cone dynamics (Morgan-Warren et al., 2013). Experimental activation of mTOR signalling in adult mice promotes RGC survival and axon regeneration after optic nerve transection (Park et al., 2008; Morgan-Warren et al., 2013). Promoting axon regeneration is relevant to scenarios in which either the optic nerve is injured, or RGCs are transplanted into the ganglion cell layer (Hertz et al., 2014) which subsequently require long distance regeneration of their axons.

Photoreceptor outer segments are damaged by light and approximately 10% of the outer segments are recycled by RPE-mediated phagocytosis each day. The digestion of internalised phagosomes is not 100% efficient and toxic lysosomal proteins such as lipofuscin, build up leading to RPE degeneration (Bharti et al., 2011). The thickening of the outer limiting membrane and successive reduction in the supply of diffusible factors to the RPE also contribute to the degeneration. The subsequent failure in photoreceptor outer segment phagocytosis by the degenerating RPE is the primary pathology in age related macular degeneration (AMD) and retinitis pigmentosa (Bharti et al., 2011).

### NTF treatment strategies

Treatments for long term RGC neuroprotection and axon regeneration are limited and delivery of individual NTF promotes incomplete and unsustained axon regeneration in the transected rat optic nerve (Logan et al., 2006) and spinal cord (Lu et al., 2004b). For example, intravitreal (*ivit*) injection of recombinant BDNF and CNTF rescues axotomised RGC from death for up to 7 days (Mey and Thanos, 1993; Ahmed et al., 2011). Long term trophic support requires repeated low dose NTF injections (Ko et al., 2000; Ko et al., 2001) since transient high peak bolus delivery of NTF down-regulates TrK receptors (Sommerfeld et al., 2000; Chen and Weber, 2004). Injectable hydrogel formulations composed of collagen, alginate or chitosan are being developed (Pakulska et al., 2012) that continuously and slowly release low titres of NTF in vivo over several weeks. However, drug loading of hydrogels is limited and thus, for chronic neurodegenerative diseases like glaucoma, sustained delivery requires repeated hydrogel implantation, making FDA approval a significant challenge. Alternative treatments, such as the transplantation of cells with extended longevity engineered to continuously produce low levels of specific NTF combinations, remove the need for repeated injections and overcome the problems with bolus NTF delivery regimes. For example, *ivit* transplantation of genetically engineered fibroblasts that overexpress fibroblast growth factor-2 (FGF-2), NT-3 and BDNF significantly increases RGC survival and axon regeneration after optic nerve crush (Logan et al., 2006).

### Stem cells and NTF treatment

Stem cells, transfected with *ntf* genes or induced to secrete NTF using epidermal growth factor (EGF)/FGF have been grafted into the retina to treat retinal degeneration e.g. : (1), BMSC secreting BDNF, glial cell line-derived neurotrophic factor (GDNF) and neurotrophin-4 are RGC neuroprotective and improve visual function in cases of traumatic optic neuropathy (Levkovitch-Verbin et al., 2010), sodium iodate-induced damage of the retina (Machalińska et al., 2013) and chronic ocular hypertension (Harper et al., 2011); (2), NSCs engineered to secrete CNTF attenuate photoreceptor death in mouse models of retinitis pigmentosa (Jung et al., 2013); (3), ESC-derived neural progenitor cells transfected with crystallin-β-b2 promote both RGC and photoreceptor survival (Bohm et al., 2012); and (4), a glucagon-like peptide-1-secreting cell line promotes RGC survival after optic nerve crush (Zhang et al., 2011). Despite possible adverse effects, cell transplantation “mono-therapies” offer the potential advantages of continuous secretion of multiple NTFs for the duration of the viability of the transplant. In the eye, BMSC/ADSC/DPSC survive for at least 3 to 5 weeks (Johnson et al., 2010; Levkovitch-Verbin et al., 2010; Haddad-Mashadrizeh et al., 2013; Mead et al., 2013) and *ivit* delivery of cell suspensions and transplantation of a retrievable permeable capsule loaded with stem cells (Zhang et al., 2011) are also viable options for patients with retinal degenerative disease (Sieving et al., 2006).

## Ivit/subretinal stem cell implantation

The fate of transplanted stem cells in the eye remains undetermined and thus the incidence of immune rejection, differentiation into unpredicted phenotypes and unbridled migration within CNS neuropil, together with possible oncogenesis, all remain poorly defined. Safeguards against these outcomes include encapsulation of the stem implant (Zhang et al., 2011) and genetic modification so that the cells carry inducible suicide genes, such as viral-derived thymidine kinase allowing selective destruction of the transplanted cells when treated with the toxic drug ganciclovir (Zhang et al., 2011). However, the potential risks of transplanting stem cells in the eye may have been exaggerated where cell movement is restrained and immune reactions muted. For example, after *ivit* injection, MSC cluster in the vitreous body (Johnson et al., 2010; Haddad-Mashadrizeh et al., 2013; Mead et al., 2013, 2013), although a small number do migrate into the retina they are neither tumorigenic nor exhibit uncontrolled growth (Johnson et al., 2010; Mendel et al., 2013; Tzameret et al., 2014). In laser-induced glaucoma and retinal injury, *ivit* BMSCs also migrate into the retina (Singh et al., 2012) where they continue to proliferate (Wang et al., 2010). After subretinal transplantation, NSCs remain immature for at least 7 months, barely proliferate and neither exhibit uncontrolled growth nor oncogenesis, but they do migrate from the injection site within the subretinal space (McGill et al., 2012; Lu et al., 2013). By contrast, after *ivit* transplantation, NSCs either attach to the retina and lens where they remain (Jung et al., 2013), or integrate into the inner retinal layers (Grozdanic et al., 2006). ESC-derived RPE cells transplanted into the subretinal space of Royal College of Surgeon (RCS) rats (which spontaneously undergo RPE and subsequent photoreceptor degeneration) survive for over 200 days, preserve visual function with evidence of neither teratoma formation (Lu et al., 2009) nor proliferation (Vugler et al., 2008). Reactive retinal gliosis rather than penetration of the internal limiting membrane is proposed as a major limitation to retinal integration of ESC after *ivit* implantation (Banin et al., 2006); whilst after subretinal grafting cell migration is more extensive (Banin et al., 2006; Lamba et al., 2009) yet still hindered by the outer limiting membrane (West et al., 2008).

## Immunological acceptance of stem cells transplanted into the eye

The vitreous cavity, like the anterior chamber of the eye, is an immunoprivileged environment (Jiang and Streilein, 1991) and thus amenable to cell transplantation. MSC fail to trigger an immune response when challenged with allogeneic lymphocytes and MSC-derived factors inhibit the proliferation of immunological cells (Kode et al., 2009; Singer and Caplan, 2011). These immunosuppressive/immunomodulatory actions of BMSC have led to Phase I (Le Blanc et al., 2004), Phase II (Le Blanc et al., 2008) and Phase III (Martin et al., 2010) clinical trials for the treatment of steroid refractory graft-versus-host disease. ADSCs suppress the immune system with the same efficacy as BMSC in vitro (Puissant et al., 2005) and increase the survival rate of transplants in animal models of graft versus host disease (Yañez et al., 2006), whereas DPSC are as efficient as BMSC in the suppression of T cell proliferation in vitro (Pierdomenico et al., 2005). Thus, the failure of the host to launch immune reactions after *ivit*/subretinal implantation of MSC is probably explained by both the immune privileged status of these sites and the immunosuppressive properties of MSC. For example, immunosuppression is not required and adverse effects are not recorded after human BMSC (Johnson et al., 2010; Levkovitch-Verbin et al., 2010; Tzameret et al., 2014)/ADSC (Haddad-Mashadrizeh et al., 2013)/rodent DPSC (Mead et al., 2013) transplantation into the eye. Equally, although not immunosuppressive, iPSC derived from the somatic cells of the recipient carry the same histocompatibility antigens and do not require immunosuppression after transplantation. By contrast, ESCs/NSCs require immunosuppression when transplanted into the CNS in animals and, since autologous transplantation is not possible, immunosuppression is required in NSC-based treatment (Cummings et al., 2005; Lu et al., 2012; Schwartz et al., 2012; Lu et al., 2013). Indeed, NSC transplantation into the subretinal space requires daily immunosuppressive treatment with cyclosporine A and dexamethasone (McGill et al., 2012). When transplanted into the vitreous without immunosuppression, NSCs are detected in just 50% of transplanted eyes 32 days after grafting (Grozdanic et al., 2006) suggesting that the immunoprivileged environment of the vitreous does not sustain survival of NSC. ESC-derived RPE cells are one of the first ESC based therapies to be used in humans and early reports of subretinal transplantation as a treatment for AMD confirm their safety, although patients require immunosuppression throughout (Schwartz et al., 2012).

## Therapeutic potential of stem cell replacement therapies

### NSC

NSC transplantation is beneficial to recovery in a range of CNS injury models, including retinal degeneration (McGill et al., 2012), SCI (Lu et al., 2003; Abematsu et al., 2010; Lu et al., 2012), stroke (Jeong et al., 2003) and TBI (Riess et al., 2002), although in many cases, it is unclear if the improved functional recovery observed is attributable to replacement of lost cells and/or trophic support of surviving cells.

For example, when transplanted into injured CNS sites such as those of SCI and TBI, NSCs differentiate into neurons and glia (Riess et al., 2002; Jeong et al., 2003; Cummings et al., 2005; Martino and Pluchino, 2006; Abematsu et al., 2010; Lu et al., 2012), replacing lost cells and providing trophic support for damaged endogenous neurons (Lu et al., 2003). NSC differentiation is greatly enhanced by containment in a matrix loaded with multiple growth factors (Lu et al., 2012) and treatment with specific differentiation factors (Cao et al., 2001). In these instances, functional recovery is attributed to the generation of new NSC-derived neurons that directly integrate into the host neuronal circuitry (Martino and Pluchino, 2006; Abematsu et al., 2010) and not to the paracrine mediated axon regeneration and neuroprotection characteristic of MSC treatment.

Despite recent success with NSC in other CNS injury models, few studies have shown the same effect in the eye. In rats, *ivit* transplantation of NSC after optic neuropathy induced by elevated intraocular pressure does not improve retinal function, despite neuronal differentiation and integration into inner retinal layers (Grozdanic et al., 2006). A similar study using mice lacking RGC (induced by removal of the superior colliculus) showed that NSCs integrate into the retina but sparsely form βIII-tubulin^+^ mature neurons and do not form functional RGC (Mellough et al., 2004). Re-innervation of central targets by the axons of replacement RGC is not yet possible and there is no evidence to suggest that regeneration of stem cell-derived RGC axons along the optic nerve occurs. Indeed, more success has been seen in RCS rats in which the retinal degeneration is of the photoreceptors rather than the RGC (McGill et al., 2012). Subretinal transplantation of NSC protects photoreceptors from death in RCS rats (McGill et al., 2012) by their phagocytosis of photoreceptor outer segments, a role usually restricted to RPE cells which, in RCS rats, are dysfunctional (Cuenca et al., 2013). Although a paracrine effect (i.e. secretion of NTF) has been suggested to mediate the effects of NSC in the retina, studies have only demonstrated this when NSCs are genetically modified (e.g. to secrete CNTF (Jung et al., 2013)). The limited number of studies published on NSC in the eye suggests that this stem cell is currently not useful for replacement of RGC, however, functional replacement of photoreceptors by NSC is more plausible because of their short synaptic distances. Despite this, integration of NSC into the outer nuclear layer (ONL) is not followed by differentiation into calbindin^+^/rhodopsin^+^ mature photoreceptors (Nishida et al., 2000) and subretinal transplanted NSCs protect, rather than replace photoreceptors (McGill et al., 2012; Jung et al., 2013).

### BMSC

In vitro neuronal differentiation and neuritogenesis of BMSC are probably artefacts resulting from cell shrinkage and toxicity yielding morphologies characteristic of neurons (Lu et al., 2004a; Neuhuber et al., 2004). Undifferentiated BMSCs co-express many functional ion channels (Li et al., 2006) as well as mature neuronal and glial markers, such as βIII-tubulin and GFAP, respectively (Karaoz et al., 2011; Tamaki et al., 2012) making successful phenotypic differentiation difficult to detect. The ability of BMSC to differentiate into neurons and replace those lost from injury is rarely reported in vivo (Vallières and Sawchenko, 2003). Their transplantation into the injury site after SCI promotes functional recovery without any evidence of neuronal replacement by BMSC differentiation (Kang et al., 2012).

In the eye, transplantation of BMSC into the vitreous after experimentally-induced glaucoma and optic nerve transection shows no evidence of their differentiation into mature retinal cells, despite some integration into the retina (Yu et al., 2006; Johnson et al., 2010; Levkovitch-Verbin et al., 2010). After transplantation into the subretinal space in RCS rats and mouse models of retinitis pigmentosa, BMSCs sparsely differentiate into cells with neuron and glia characteristics, but not mature photoreceptors or RPE cells (Zhang and Wang, 2010; Tzameret et al., 2014) and a protective effect on endogenous photoreceptors and RPE cells is observed (Arnhold et al., 2007; Lu et al., 2010).

### ADSC

There is conflicting evidence for the differentiation of ADSC into neurons in vivo and in vitro (Anghileri et al., 2008; Ye et al., 2010). BDNF/retinoic acid treatments induce the differentiation of ADSC into functional neurons, confirmed by patch clamp analysis and the expression of phenotypic neuronal markers (Anghileri et al., 2008). The ADSC-derived neuronal phenotypes demonstrated in this and other studies (Ye et al., 2010) is only transient with de-differentiation occurring after withdrawal of the differentiation-inducing medium (Ye et al., 2010), explaining why ADSC-derived neurons are rarely seen in vivo after transplantation in animal models of stroke (Kang et al., 2003). Both studies (Kang et al., 2003; Ye et al., 2010) concluded that cerebrospinal fluid and CNS neuropil do not sustain neuronal differentiation of ADSC. By contrast, ADSCs pre-differentiated into NG2^+^/S100^+^ glia survive for up to 8 weeks after transplantation into rodent SCI sites (Arboleda et al., 2011) and thus, like other MSC, probably differentiate preferentially into glia in vivo (Cao et al., 2001; Cho et al., 2009; Leong et al., 2012).

ADSCs survive for up to 90 days in the vitreous cavity after transplantation although their fate has not been studied (Haddad-Mashadrizeh et al., 2013). Interestingly, ADSC transplanted into the vitreous cavity of mouse models of diabetic retinopathy preferentially differentiate into pericytes, associating with and conserving the retinal vasculature, suggesting a unique role for ADSC in treating diabetic retinopathy (Mendel et al., 2013). The failure of ADSC to integrate into the retinal layers diminishes their potential for RGC and photoreceptor replacement (Mendel et al., 2013).

### DPSC

DPSC differentiate into functionally active neurons in vitro (Arthur et al., 2008; Kiraly et al., 2009) and, when transplanted, integrate and survive in injured rat brain tissue for at least 4 weeks (Kiraly et al., 2011; Fang et al., 2013). Other studies demonstrate that, although DPSC-derived neurons express neuronal phenotypic markers, they neither generate action potentials nor form functional neuronal networks (Aanismaa et al., 2012). Like BMSC, they constitutively express mature neuronal and glial phenotypic markers even in an undifferentiated state and this may explain the contradictions in the literature if these characteristics are taken as a read out of successful differentiation (Karaoz et al., 2011; Tamaki et al., 2012). In vivo, transplantation of DPSC into rat SCI lesion sites leads to functional recovery yet only glial, not neuronal, differentiation is observed (Sakai et al., 2012), suggesting that differentiation of DPSC into neurons is possible in vitro but currently has not yet been realised in vivo. After transplantation into the vitreous, DPSC do not differentiate into neurons and fail to integrate into the retina (Mead et al., 2013), limiting their potential as a cell replacement therapy.

### ESC/iPSC

**Figure 2:**
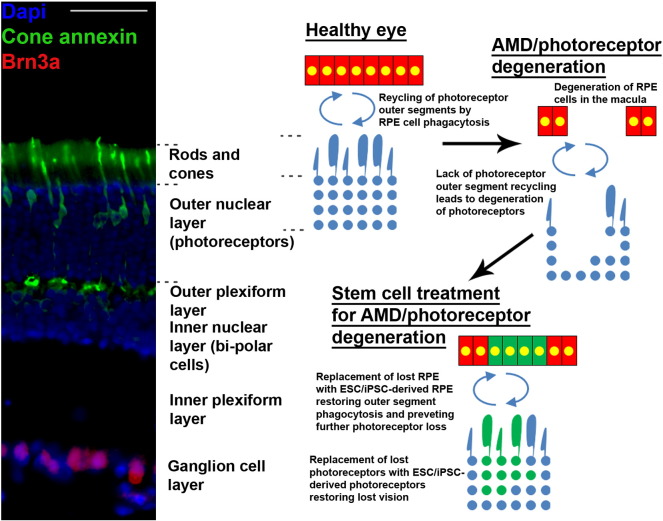
A diagram showing the proposed way in which ESC/iPSC-derived RPE and photoreceptors can be used to treat AMD/photoreceptor degeneration. The left panel shows a rat retina immunohistochemically stained for cone annexin (cone photoreceptor marker; green), Brn3a (RGC marker; red) and DAPI (nuclear marker; blue) with the individual layers labelled (scale bar: 100 μm). On the right, RPE is represented together with photoreceptor loss in AMD and the potential for cell replacement in preventing visual decline and restoring vision.

The greatest potential for cell replacement has been seen with ESC/iPSC, which can be successfully predifferentiated prior to transplantation in the eye, with the most success demonstrated in RPE/photoreceptor replacement for AMD (Fig. 2).

ESC can be directed towards a retinal phenotype with developmental induction signals including bone morphogenetic protein (BMP) antagonists (Lamb et al., 1993), Wnt inhibition (Wilson and Houart, 2004) and insulin-like growth factor (IGF) treatment (Pera et al., 2001). Accordingly, 30% of ESC/iPSC differentiate into retinal progenitors (Ikeda et al., 2005), a number that increases to 80% for both ESC (Lamba et al., 2006) and iPSC (Tucker et al., 2011) by incorporating BMP/Wnt inhibition with IGF and FGF treatments. These ESC/iPSC-derived retinal progenitors successfully mature into photoreceptors as well as RPE cells and integrate into retinal explants after co-culture with adult retina/retinal neurons (Osakada et al., 2008) or after the addition of a cocktail of small molecules (Osakada et al., 2009b). Comparisons of the gene expression profiles of ESC-derived retinal cells with primary developing foetal retinal cells using microarray analysis show them to be highly conserved between the two cell sources throughout development (Lamba and Reh, 2011).

ESC-derived retinal progenitors, primed to form neuronal retina rather than RPE using FGF, successfully differentiate into photoreceptors (Hambright et al., 2012), integrate into the ONL (Lamba et al., 2009) and survive for over 3 months in the subretinal space of non-immunosuppressed mice with an intact blood–retinal barrier, with integration more significant when the retina is injured (Hambright et al., 2012). iPSC-derived photoreceptors transplanted into the subretinal space integrate into the ONL and increase retinal function as determined by electroretinogram (ERG) (Tucker et al., 2011). Transplantation of ESC-derived photoreceptors into the vitreous of newborn mice leads to their correct topographic integration into all the layers of the retina, i.e. ESC-derived photoreceptors move to the ONL, whereas ESC-derived amacrine cells and RGC-like cells migrate to the inner nuclear layer/ganglion cell layer (Lamba et al., 2009; Reynolds and Lamba, 2013). However, integration is only possible up to 48 h after birth, corroborating reports that in adult rats, *ivit* ESC-derived cells fail to integrate into the retina (Banin et al., 2006).

Both mouse (Eiraku et al., 2011) and human (Nakano et al., 2012) ESC can be induced to form a complete topographically organized retina, including the RPE. Developing photoreceptors, isolated from ESC-derived ex vivo retina, integrate after transplantation into mouse models of retinal degeneration (Gonzalez-Cordero et al., 2013). These findings have been replicated using iPSC showing the formation of a synaptically connected stratified retina (Phillips et al., 2012).

ESC/iPSC can be induced to predominantly differentiate into RPE cells using similar protocols as above, but with the omission/antagonism of FGF to bias the generation of RPE cells over neural retina (Meyer et al., 2009; Osakada et al., 2009a). These ESC/iPSC-derived RPE cells phagocytise photoreceptor outer segments (Carr et al., 2009a) and preserve retinal function in the RCS rats (Vugler et al., 2008; Carr et al., 2009b). A study comparing adult human ESC-derived RPE with foetal human RPE demonstrated a strong correlation in their gene expression profiles. However, iPSC-derived RPE have a distinct gene expression profile, indicating potential differences between ESC-derived retinal cells and iPSC-derived retinal cells (Liao et al., 2010).

Subretinal transplantation of ESC/iPSC-derived RPE in cases of AMD requires approximately 60,000 cells (Bharti et al., 2011) to restore RPE-mediated recycling of photoreceptor outer segments. In contrast to photoreceptor replacement, in this instance significant migration, integration and synaptogenesis is not required to achieve functional efficacy. Its effectiveness is already proven by the fact that current surgical intervention relies on the same principles i.e. translocating the macula to an adjacent, healthy portion of RPE (da Cruz et al., 2007). These attributes have led to the first clinical trial transplanting ESC-derived RPE cells in patients with AMD (Schwartz et al., 2012).

ESCs/iPSCs are able to differentiate into RGC and, during the formation of ESC-/iPSC-derived retina ex vivo, RGCs are the first cells to develop which mimic normal retinal development (Eiraku et al., 2011; Nakano et al., 2012; Phillips et al., 2012). The yield of RGC is enhanced by transfection of the stem cells with genes regulating RGC development, namely *math5* and *sox4* (Jiang et al., 2013). Similar to ESC/iPSC-derived photoreceptors integrating into the ONL, transplanted adult rat RGCs integrate and survive in the ganglion cell layer (Hertz et al., 2014) but, unlike photoreceptors, the long distances over which RGC axons must regenerate to re-innervate central targets is unachievable (Sun et al., 2011).

## Therapeutic potential of stem cell trophic support (Fig. 1; Table 2)

### NSC

When transplanted into SCI lesion sites, NSCs increase the expression of NGF, BDNF, NT-3 and GDNF within the lesion site (Gu et al., 2012; He et al., 2012) and promote axonal sprouting (Lu et al., 2003). However, the trophic support provided by undifferentiated NSC only minimally restores function compared to when they are induced to differentiate down a neuronal lineage before or after transplantation into SCI sites (Cao et al., 2001; Abematsu et al., 2010; Gu et al., 2012; He et al., 2012). In the eye, as stated above, *ivit* NSCs transplanted into the vitreous fail to improve function in models of elevated intraocular pressure-induced RGC loss (Grozdanic et al., 2006) and axotomy (Flachsbarth et al., 2014) and only show neuroprotective efficacy when transfected to secrete CNTF. However, transplantation was made four weeks post-injury, so that it cannot be ruled out that NSC may be able to have a paracrine-mediated neuroprotective effect on RGC if they were transplanted at the time of injury when injured RGCs are most amenable to neuroprotective strategies. Nonetheless, after subretinal transplantation of NSC into RCS rats, rather than replaced, photoreceptors are protected against death by NSC-directed phagocytosis of photoreceptor outer segments (Cuenca et al., 2013) and induction of CNTF expression by Müller glia (Lu et al., 2013).

### BMSC

The neurotrophic secretome of BMSC, which includes NGF, BDNF, NT-3, NT4/5, CNTF, GDNF and PDGF is widely documented (Dormady et al., 2001; Chen et al., 2005; Wilkins et al., 2009; Ghorbanian et al., 2012; Sakai et al., 2012; Johnson et al., 2013; Mead et al., 2013; Mead et al., 2014) and places them as a candidate cellular therapy to combat ocular neurodegeneration. BMSC-mediated neuroprotection of RGC is reported to be mediated by PDGF (Johnson et al., 2013), whilst other studies have shown that BMSC-induced RGC neuroprotection and axon/neurite growth is mediated by NGF, BDNF and NT-3 (Mead et al., 2013). The importance of BMSC-derived NTF for retinal neuron survival is confirmed by using TrK and PDGFR inhibitors which significantly diminish the RGC neuroprotection and/or neurite growth effects elicited by BMSC (Johnson et al., 2013; Mead et al., 2013; Mead et al., 2014). The vitreous does not permit the differentiation of BMSC into neurons (Hill et al., 2009). Nonetheless, *ivit* transplanted BMSCs secrete diffusible NTF, BDNF and NT-3 (Mead et al., 2013), directly protecting RGC from death in animal models of glaucoma (Yu et al., 2006; Johnson et al., 2010) and optic nerve transection (Levkovitch-Verbin et al., 2010; Mead et al., 2013), and can also be indirectly effective by inducing Müller cell NTF production (Lee et al., 2012). Interestingly, BMSC also promote the regeneration of RGC axons after optic nerve crush (Mead et al., 2013), probably through the same NTF-mediated mechanisms (Berry et al., 2008). Subretinal and *ivit* BMSC transplantation in RCS rats and mouse models of retinitis pigmentosa significantly improves retinal function by preserving photoreceptor and RPE cell viability (Arnhold et al., 2007; Lu et al., 2010; Tzameret et al., 2014) and, although the underlying observations remain equivocal, a role for the NTF secretome in promoting cell survival is a likely explanation.

### ADSC

ADSCs express NGF, BDNF, NT-3, GDNF, VEGF and PDGF (Kalbermatten et al., 2011; Zhou et al., 2013; Mead et al., 2014), with titres of BDNF and vascular endothelial growth factor (VEGF) being significantly higher than those secreted by BMSC (Zhou et al., 2013). Despite this, ADSCs are relatively untested in the eye but have efficacy as a paracrine-mediated therapy in other CNS animal injury models like SCI (Arboleda et al., 2011; Zhou et al., 2013) and stroke (Kang et al., 2003). In co-culture, ADSC-derived NTF promote neuroprotection and neuritogenesis of injured RGC, although the effects are not as pronounced as those achieved with BMSC/DPSC (Mead et al., 2014). In a mouse model of light induced photoreceptor damage, both *ivit* ADSC and ADSC-conditioned medium preserve ONL thickness and the amplitude of the a-wave of the ERG (Sugitani et al., 2013; Tsuruma et al., 2014). Progranulin, tissue inhibitor of metalloproteinases-1 (TIMP1) and the secreted protein rich in cysteine (SPARC) are the active agents produced by ADSC in vitro and, after *ivit* transplantation, have similar effects to *ivit* ADSC/ADSC conditioned medium. Together, these data suggest that ADSC have therapeutic potential for neurodegenerative conditions through NTF production, with many of the active factors different from those produced by BMSC and DPSC.

### DPSC

Like other MSCs, DPSCs have an extensive neurotrophic secretome which includes NGF, BDNF, NT-3, GDNF, VEGF and PDGF (Nosrat et al., 1997, 2001; Gale et al., 2011; Sakai et al., 2012; Mead et al., 2013, 2014). Interestingly, DPSCs express significantly greater amounts of *ngf*, *bdnf* and *nt-3* mRNA than BMSC (Sakai et al., 2012) and this is true also for the secreted proteins NGF, BDNF and NT-3 (Mead et al., 2013). DPSC-conditioned medium containing the above factors promotes neurite outgrowth of cortical neurons (Sakai et al., 2012), a neuroblastoma cell line (Ishizaka et al., 2013) and primary RGC (Mead et al., 2013, 2014) with significantly greater efficacy than BMSC and ADSC-conditioned medium. DPSCs transplanted into mouse hippocampus increase the basal expression levels of many NTF such as CNTF, VEGF, FGF-2 and NGF (Huang et al., 2008), although it is unknown if the transplanted DPSCs directly express these NTFs and/or indirectly promote the expression of NTFs by neighbouring cells in the surrounding neuropil. DPSC transplantation into rat SCI lesion sites leads to greater functional improvement than BMSC transplantation and, with a lack of observable neuronal differentiation, the evidence strongly suggests a paracrine-mediated mechanism (Sakai et al., 2012). Following either *ivit* transplantation or co-culture with injured RGC, DPSCs secrete NGF, BDNF and NT-3 and promote RGC survival and axon/neurite regeneration; effects which are attenuated by Fc-TrK blockers (Mead et al., 2013, 2014). These neuroprotective/pro-regenerative effects are significantly greater in DPSC transplanted animals compared to BMSC transplanted animals and are correlated with a more favourable neurotrophic secretome by DPSC compared to BMSC (Mead et al., 2013, 2014). Currently, no evidence exists for DPSC-mediated protection of photoreceptors whilst further research into the mechanisms of DPSC-mediated RGC neuroprotection is required.

### ESC/iPSC

Unlike MSC, the paracrine potential of ESC/iPSC for treating the injured retina/CNS is as yet unknown. Addition of TrK receptor blockers to ESC cultures perturbs their survival, indicating that neurotrophins are released and active in an autocrine fashion, but further analysis on the secretome is required (Pyle et al., 2006). *ivit* transplantation of ESC-derived photoreceptors promotes the survival of nearby endogenous photoreceptors (Meyer et al., 2006). Similarly it is known that RPE cells secrete VEGF and PEDF, which may further explain how ESC-derived RPE cells protect photoreceptors from death (Strauss, 2005).

## Conclusions

The use of stem cells has proven potential as a cellular therapy for retinal degenerative conditions through replacement of lost cells in the eye and/or the release of growth factors into damaged neuropil. However, the mechanism of action as well as the efficacy of the cellular therapy vary between different stem cells and can contrast greatly with what is seen in other models of CNS injury. ESCs/iPSCs have shown potential as a source of retinal cells for replacement of particularly photoreceptors and RPE, but their possible paracrine action is currently not known. Although the potential trophic properties are still not fully understood, NSCs have proven impressive cell replacement properties in other CNS regions and these faculties may be enhanced, optimised and refined by pre-treatment with selected growth/inducible factors leading to their formulation as an effective cell replacement therapy in the retina. By contrast the dominant mechanism by which MSCs restore lost retinal function appears to be paracrine-mediated, which offers the potential for their use to provide continuous delivery of multiple growth factors to provide direct trophic support for neurons in the degenerate retina and to stimulate glia to indirectly help effect neural repair. The non-invasive, non-tumorigenic, immunosuppressive and trophic characteristics of MSC, along with the relatively ease of access from their diverse adult tissue sources, circumvent moral and ethical dilemmas and make the autologous and allogeneic intra-ocular implantation of MSC a promising paracrine-mediated therapy for the diseased eye.

## Author contributions

Ben Mead: conception and design; collection and/or assembly of data; data analysis and interpretation; manuscript writing.

Martin Berry: manuscript writing; final approval of manuscript.

Ann Logan: conception and design; data analysis and interpretation; manuscript writing; final approval of manuscript.

Robert Scott: final approval of manuscript.

Wendy Leadbeater: conception and design; data analysis and interpretation; manuscript writing; final approval of manuscript.

Ben A. Scheven: conception and design; data analysis and interpretation; manuscript writing; final approval of manuscript.

## Figures and Tables

**Table 1:** Current clinical trials that test the safety and efficacy of stem cell transplantation for the treatment of degenerative eye disease. Further details found at www.clinicaltrials.gov.

Treatment	Disease	Stage	No. of subjects	Estimated completion date	Outcome	Clinicaltrials.gov identifier
Intravitreal BMSC	AMD, glaucoma	Recruiting participants	300	Aug 2017	Visual acuity, visual field	NCT01920867
Intravitreal BMSC	AMD, diabetic retinopathy, retinitis pigmentosa	Recruiting participants (Park et al., 2015)	15	Dec 2015	Incidence and severity of adverse events	NCT01736059
Intravitreal BMSC	Retinitis pigmentosa	Recruiting participants	10	Aug 2016	Visual acuity, quality of life, visual field, ERG, VEP, colour vision, contrast sensitivity	NCT02280135
Intravitreal BMSC	Glaucoma	Recruiting participants	10	Dec 2016	Incidence and severity of adverse events, visual acuity, visual field, OCT, ERG	NCT02330978
Intravitreal BMSC	Retinitis pigmentosa	Completed (Siqueira et al., 2011)	50	June 2013	Visual acuity	NCT01560715
Intravitreal BMSC	Ischemic retinopathy	Recruiting participants	30	Jan 2014	Size of foveal avascular zone	NCT01518842
Intravitreal BMSC	AMD	Recruiting participants	1	June 2015	Incidence and severity of adverse events	NCT02016508
Intravitreal BMSC	AMD, Stargardt's macular dystrophy	Recruiting participants	10	Dec 2015	Visual acuity	NCT01518127
Intravenous bone marrow mononuclear cells	Optic atrophy	Recruiting participants	24	July 2016	Visual function, reduction in optic nerve degeneration	NCT01834079
Intravitreal AMSC	Dry AMD	Recruiting participants	100	June 2016	Incidence and severity of adverse events, visual acuity	NCT02024269
Subretinal ESC-derived RPE	Dry AMD	Recruiting participants	12	April 2016	Visual acuity, ERG, OCT	NCT01674829
Subretinal ESC-derived RPE	AMD	Pre-recruitment	10	June 2017	Incidence and severity of adverse events, visual acuity	NCT01691261
Subretinal ESC-derived RPE	Stargardt's macular dystrophy	Recruiting participants (Schwartz et al., 2012; Schwartz et al., 2014)	16	Dec 2014	Incidence and severity of adverse events	NCT01345006
Subretinal ESC-derived RPE	Dry AMD	Recruiting participants Schwartz et al. (2014)	16	Dec 2014	Incidence and severity of adverse events	NCT01344993

**Table 2:** NTF known to be secreted by NSC, BMSC, ADSC and DPSC. NTF secretion by ESC/iPSC is currently unreported.

Stem cells	Neurotrophic factor secretion profile
NSC	NGF, BDNF, NT-3, GDNF (Lu et al., 2003); (Gu et al., 2012); (He et al., 2012)
BMSC	NGF, BDNF, NT-3, NT-4/5, CNTF, GDNF, PDGF (Dormady et al., 2001); (Chen et al., 2005); (Wilkins et al., 2009); (Ghorbanian et al., 2012); (Sakai et al., 2012); (Johnson et al., 2013); (Mead et al., 2013, 2014)
ADSC	NGF, BDNF, NT-3, GDNF, VEGF, Progranulin, SPARC (Kalbermatten et al., 2011); (Sugitani et al., 2013); (Zhou et al., 2013); (Tsuruma et al., 2014); (Mead et al., 2014)
DPSC	NGF, BDNF, NT-3, CNTF, GDNF, VEGF, FGF-2 (Nosrat et al., 1997, 2001); (Huang et al., 2008); (Gale et al., 2011); (Sakai et al., 2012); (Mead et al., 2013, 2014)
